# MicroRNA genes preferentially expressed in dendritic cells contain sites for conserved transcription factor binding motifs in their promoters

**DOI:** 10.1186/1471-2164-12-330

**Published:** 2011-06-27

**Authors:** Bastiaan JH Jansen, Iziah E Sama, Dagmar Eleveld-Trancikova, Maaike A van Hout-Kuijer, Joop H Jansen, Martijn A Huynen, Gosse J Adema

**Affiliations:** 1 Department of Tumor Immunology, Nijmegen Centre for Molecular Life Sciences, Radboud University Nijmegen Medical Centre, Nijmegen, the Netherlands; 2 Centre for Molecular and Biomolecular Informatics, Nijmegen Centre for Molecular Life Sciences, Radboud University Nijmegen Medical Centre, Nijmegen, the Netherlands; 3 Central Hematology Laboratory, Radboud University Nijmegen Medical Centre, Nijmegen, the Netherlands

## Abstract

**Background:**

MicroRNAs (miRNAs) play a fundamental role in the regulation of gene expression by translational repression or target mRNA degradation. Regulatory elements in miRNA promoters are less well studied, but may reveal a link between their expression and a specific cell type.

**Results:**

To explore this link in myeloid cells, miRNA expression profiles were generated from monocytes and dendritic cells (DCs). Differences in miRNA expression among monocytes, DCs and their stimulated progeny were observed. Furthermore, putative promoter regions of miRNAs that are significantly up-regulated in DCs were screened for Transcription Factor Binding Sites (TFBSs) based on TFBS motif matching score, the degree to which those TFBSs are over-represented in the promoters of the up-regulated miRNAs, and the extent of conservation of the TFBSs in mammals.

**Conclusions:**

Analysis of evolutionarily conserved TFBSs in DC promoters revealed preferential clustering of sites within 500 bp upstream of the precursor miRNAs and that many mRNAs of cognate TFs of the conserved TFBSs were indeed expressed in the DCs. Taken together, our data provide evidence that selected miRNAs expressed in DCs have evolutionarily conserved TFBSs relevant to DC biology in their promoters.

## Background

In recent years, microRNAs (miRNAs) have taken center stage, as they are key regulators of gene expression at the post-transcriptional level, and play a fundamental role in a wide variety of biological processes, such as cell growth, development and several pathological conditions [1-3]. MicroRNAs are small, ~22 nt long, single-stranded molecules which, when complexed with an RNA-induced silencing complex (RISC), are able to form a complementary double-stranded RNA structure by hybridizing to the 3' untranslated region of target transcripts, and inhibit translation of their cognate mRNA and/or promote their degradation [4]. MicroRNAs have an established role in hematopoietic development and immunity. For example, forced expression of miR-181 in hematopoietic progenitors leads to an increase in the number of B cells [5], whereas it sets T cell receptor signaling thresholds by targeting negative regulators [6]. MicroRNA-146a is up-regulated during toll-like receptor (TLR) signaling and targets TNF receptor-associated factor 6 (TRAF6) and IL-1 receptor-associated kinase 1 (IRAK1) [7], thereby serving in a negative feedback loop. Moreover, miR-155 is also up-regulated during TLR and TNF signaling [8], and is required for normal immune function [9-14].

Although great strides have been made towards understanding the biogenesis of miRNAs [4] and the identification of mRNA targets [15], their own expression is one of the least understood aspects. They are transcribed by RNA polymerase II [16] or RNA polymerase III [17]. In addition, approximately 80% of miRNAs are located in introns of protein coding genes, but at least one third is believed to be transcribed independently from their host gene [4,18-20], whereas recent data suggest that most, if not all, intronic miRNAs contain putative promoters independent of their host gene [21]. In fact, it is now believed that once physically accessible, a gene is regulated by transcription factors that bind to their cognate transcription factor binding site (TFBS) in its promoter. Usually, there is more than one TFBS per gene, allowing combinations of transcription factors to elicit gene transcription. This phenomenon has been predicted for instance in *Plasmodium falciparum*, a parasite with a dearth of transcription-associated factors [22-24] and has been experimentally validated in other eukaryotic promoters [25,26].

Myeloid dendritic cells (DCs) and monocytes arise from a common monocyte/dendritic cell progenitor [27]. *In vitro*, DCs can be generated from blood-derived monocytes when cultured in the presence of the cytokines *interleukin 4 *(IL4) and *granulocyte/macrophage colony stimulating factor *(GM-CSF) [28]. DCs play an important role in innate immunity and the initiation of adaptive immune responses. They capture foreign antigens in peripheral tissues, migrate to the T-cell areas of secondary lymphoid organs and present these antigens to T- and B-cells. Depending on the extracellular signals they receive, they either induce tolerance in the steady state (tolerogenic DCs), or an inflammatory response in the presence of pathogen-associated patterns (PAMPs) or inflammatory cytokines (activated or mature DCs) [29,30]. As a consequence, DCs have gained considerable interest as vaccine adjuvants and are currently exploited in the treatment of cancer after loading with tumor-cell derived antigens [31,32].

In order to gain insight in miRNAs that may regulate DC development and behaviour, expression profiles of 157 miRNAs were obtained from monocytes and DCs under inflammatory and tolerizing conditions. We show that DCs express a wide variety of miRNAs, some of which are differentially regulated during DC development and maturation. We predicted several target genes for these miRNAs, as well as binding sites for transcription factors in the putative promoter regions of these miRNAs. Furthermore, we show that by also taking evolutionary conservation [33,34] of the identified TFBSs into account, binding sites were found to preferentially cluster within 500 bp upstream of the pre-miRNAs. Also, the fraction of conserved TFBSs for which the cognate transcription factors are expressed in DCs increases with the number of miRNA promoters that contain these TFBSs. Taken together, the data described here provide evidence that the promoter regions of the miRNAs expressed in myeloid DCs contain binding sites for motifs of transcription factors that are relevant to myeloid cell biology. This may help expand the understanding of the molecular mechanisms underlying DC biology and development.

## Methods

### Isolation, culture and characterization of monocytes and DCs

Peripheral blood mononuclear cells (PBMCs) were isolated as previously described [28]. Monocytes were isolated from PBMCs using CD14+ selection and the AutoMACS technology (Miltenyi Biotec, Bergisch Gladbach, Germany) following the manufacturer's directions. Human monocyte-derived DCs were generated using GM-CSF and IL-4 and matured as described previously. Purity and maturation of monocytes and DCs were assessed by means of FACS analysis as described previously [28]. Mixed lymphocyte reactions were essentially carried out as described elsewhere [35]. Briefly, they were co-cultured with peripheral blood lymphocytes in various dilutions for 2 to 4 days. Then tritiated thymidine (1 μCi/well; MP Biomedicals) was added to the cell cultures and incorporation was measured after 16 hr. Enzyme-linked immunosorbent assays (ELISA) were performed to assess the secretion of the inflammatory cytokines TNFα and IL8, using BD OptEIA (BD Biosciences, San Jose, CA) kits following the manufacturer's recommendations.

### Isolation of total RNA and microRNA-specific reverse transcription and quantitative PCR

Total RNA was isolated using TRIzol (Invitrogen, Carlsbad, CA), following the manufacturer's recommendation. Quantity and purity were determined spectrophotometrically. For each miRNA, 4 ng of total RNA was used as input and miRNAs were converted to cDNA using the TaqMan miRNA Reverse Transcription cDNA Synthesis kit and miRNA-specific looped primers from the Early Access miRNA Profiling Kit (both from Applied Biosystems, Foster City, CA) following the manufacturer's recommendations. Real-time quantitative PCR was performed using miRNA-specific primer/probe pairs from the Early Access miRNA Profiling Kit and reactions were carried out as described elsewhere [36]. Actual amplification and data collection were performed on the ABI 7700 Sequence Detection System (Applied Biosystems, Foster City, CA). After visual inspection, data were exported to a text file and further analyzed in Microsoft Excel (Microsoft Corporation, Redmond, WA) and various Bioconductor packages [37] in the R statistics environment [38].

### Statistics for miRNAs expression data

Delta Ct values for each miRNA were calculated with *hsa*-let-7a as a reference, according to the manufacturer's directions. Only miRNAs with a dCt ≤ 12 (where dCt = Ct_target _- Ct_*hsa*__-let-7a_) were considered for further analysis. Data were combined into a convenient *ExpressionSet *(eSet) structure using the Biobase package, and further analyzed by means of gene-by-gene one-factor ANOVA using the package LMGene, all in Bioconductor and R. Only genes that had a False Discovery Rate-adjusted p-value of < 0.05 were considered differentially expressed. These genes were further assessed using non-parametric pairwise comparison of the different myeloid cell subsets using Tukey's post-hoc test in the R statistics package. Genes with a p-value < 0.05 were considered differentially expressed.

### Determination of TFBSs in microRNA promoters

Putative promoter regions extending 2 kb upstream of the miRNAs were extracted from the Genome Browser sno/miRNA track of the UCSC March 2006 human genome assembly [39,40]. The program Clover [41] was used to screen for over-represented TFBSs in these sequences using a precompiled library of TFBS motifs. The library contained 263 TFBS motifs (position-specific weight matrices) constructed from the JASPAR core database (2005) [42] and TRANSFAC version 7.0 [43].

To determine over-represented TFBS motifs, Clover starts for every location in a DNA sequence, by calculating a score reflecting the likelihood that a certain TF binds at that location. This score is a likelihood ratio, with in the numerator the probability that the sequence matches the positional weight matrix of the motif (the product of the frequencies of the nucleotides in the weight matrix at the positions corresponding to those in the sequence) and in the denominator the likelihood that the sequence is derived from a random sequence (the product of the frequencies of the nucleotides in each position in the background sequence). This likelihood ratio is then averaged per complete sequence and over all subsets of the set of sequences. Finally a "raw score" is derived, by taking the logarithm of the averaged likelihood ratio. A raw score above zero thus signifies over-represented motifs and a raw score below zero signifies under-represented motifs. The raw score increases when more of the sequences contain good motif matches, and also when there are more good matches per sequence. The p-values for over-represented motifs are subsequently derived by analyzing, whether random sets of promoter sequences from all the genes in the genome are likely to have the same, or a higher "raw score". Clover examines the over-representation of multiple TFBSs and takes into account the multiple testing issue for its p-value calculation. Besides the raw score, Clover also reports an instance score, which is the logarithm of the likelihood ratio mentioned above for any specific TFBS at any specific position in the sequence.

Our inputs to Clover are the miRNA promoter sequences and the 263 TFBS motifs mentioned above. For statistical calculations, promoter regions 2 kb upstream of all human genes in the genome are included as background. Our thresholds for Clover outputs are instance score ≥ 6 for recognizing a specific TFBS, and p-value ≤ 0.05 for over-represented motifs (default values in Clover). In addition, any mention of a TF motif score in subsequent sections refers to the instance score for the TFBS at a specific location in a promoter sequence. Our motivation for including instance scores is to enable us to calculate the conservation of the nucleotides at high-scoring TFBSs.

### Calculation of evolutionary conservation score of TFBSs

In order to take into account conservation of TFBSs in the miRNA promoters, the PhastCons conservation track from the UCSC Genome Browser of January 2009 (http://hgdownload.cse.ucsc.edu/goldenPath/hg18/phastCons44way/) was used, which represents the conservation of each nucleotide across 44 placental mammals as calculated using the PhastCons program [33]. The mammalian conservation track was used because we examined the expression of mammalian miRNAs that are generally not conserved outside this clade [44,45]. The base-by-base conservation scores are derived from a two-state phylo-HMM and are defined as the posterior probability that the corresponding alignment column was generated by the conserved state and not the non-conserved state of the phylo-HMM used in the calculation [33]. The score ranges from 0 to 1; the higher the value, the more conserved the nucleotide. Using in-house Python scripts, we obtained the conservation score for a TFBS as the median of the PhastCons scores of the bases in that TFBS.

### Microarray sample preparation of DCs and microarray analysis

From one donor, 3 different samples (1 × 10^7 ^cells; technical replicate) were generated per DC subtype (immature monocyte-derived DCs, maturing DCs, tolerogenic DCs and activated tolerogenic DCs) and RNA for microarray analysis was isolated using the RNeasy Total RNA Extraction kit (Qiagen, Venlo, the Netherlands). Quality control, conversion to labeled RNA, hybridization and scanning were all performed at the microarray facility of the Department of Human Genetics (Radboud University Nijmegen Medical Centre Nijmegen, Nijmegen, the Netherlands), using Affymetrix technology while following the manufacturer's protocols. The .CEL files were processed using the Bioconductor package limma in the R statistics environment and further analyzed with the package panp to generate presence/absence calls from the microarray data. In this study, intensities above the p-value cut-off of 0.01 indicated presence, p-values between 0.01 and 0.02 indicated marginal presence, and p-values above 0.02 indicated absence. Complete, MIAME-compliant datasets were deposited with the Gene Expression Omnibus of the National Center for Biotechnology Information (http://www.ncbi.nlm.nih.gov/geo/) and can be accessed through GEO Series accession number GSE23371.

### Analysis of miRNA targets

In addition to identifying miRNAs that are expressed in DCs and monocytes, it is important to identify some of their target genes. This may help in understanding the miRNA-related molecular mechanisms underpinning DC maturation. We used TargetScanHuman version 5.1 [15] to predict the target genes for the miRNAs identified to be expressed in DCs and monocytes. The precompiled predictions were downloaded from the official TargetScan website (http://www.targetscan.org/) and were further filtered for RefSeq transcripts having at least one site that is conserved across placental mammals and also had a target score (total context score) of ≤ -0.4 before further analyses. To improve the reliability of the target genes predicted, we used a second miRNA target prediction tool, PicTar [46]. The precompiled miRNA target predictions were downloaded from the PicTar website at http://pictar.mdc-berlin.de, on January 2011. We filtered the predictions at a PicTar threshold of ≥0.4. For further analysis we used the target genes that were predicted by both TargetScan and PicTar and also by each of the algorithms separately. To narrow the scope of the target genes to DCs, we used the previously published dataset of Lehtonen and colleagues wherein the relative expression levels of gene expression in DCs relative to monocytes are reported [47]. Using their data, we selected genes that are at least 2-fold down-regulated in DCs relative to monocytes and that we also predicted to be target genes for DC-expressed miRNAs. Comparisons between our predicted target genes and genes that were over- or under-expressed in DCs relative to monocytes in the Lehtonen dataset were done at the level of RefSeq DNA ID to ensure that the correct gene isoforms were being matched. (The Lehtonen dataset was based on global gene expression analyses using Affymetrix HG-U133A Gene Chip oligonucleotide arrays. We used Biomart in Ensembl version 62 available at http://www.ensembl.org/biomart to obtain the corresponding RefSeq DNA IDs for the probesets in the microarray dataset). The set of genes so selected are likely to be important in miRNA regulation of DC maturation from monocytes.

### Gene Ontology analysis

Gene ontology enrichment analyses were done using the Cytoscape [48] plugin BINGO version 2.3 [49]. Using this software, we tested for over-representation using the hypergeometric test with Benjamini & Hochberg False Discovery Rate (FDR) correction. GO processes reported were deemed significant when the corrected p-value was < 0.05. Venn diagrams were drawn using the Venny tool of Oliveros, J.C. available at http://bioinfogp.cnb.csic.es/tools/venny/index.html.

## Results

### Monocytes and monocyte-derived dendritic cell subtypes express distinct microRNAs

Monocytes were isolated to > 90% purity and monocyte-derived dendritic cell subtypes were generated and validated as depicted in Additional file 1, Figure S1. A TaqMan-based quantitative RT-PCR method was used to profile the expression of miRNAs. Immature monocyte-derived DCs (iDCs) were induced to either undergo maturation by LPS stimulation for 6 hr (mDCs), to mature into tolerogenic DCs (tDCs) in the presence of IL-10 and dexamethasone for 24 hr, or to mature into activated tolerogenic DCs (atDCs; previously described by Emmer *et al. *[50]) in the presence of IL-10 and dexamethasone for 24 hr, followed by 6 hr of LPS (Additional file 1, Figure S1A). Phenotypical and functional characterization confirmed that mature DCs expressed high levels of CD80 and CD86 (Additional file 1, Figure S1B), induced strong proliferation of allogeneic T cells in a mixed lymphocyte reaction (Additional file 1, Figure S1C) and secreted high levels of pro-inflammatory cytokines TNF and IL8 (Additional file 1, Figure S1D). Accordingly, iDCs and tDCs did not show up-regulation of CD80 and CD86, induced much less proliferation and produced less cytokines. Activated tolerogenic DCs showed a more intermediate pattern of activation, indicative of less profound TLR signaling in response to LPS. The expression profile of 157 miRNAs in monocytes and the various DC activation stages was determined in a primary screen involving total RNA from one donor as shown in Additional file 2, Figure S2.

Of the 157 miRNAs screened, 104 were detected and expressed to variable degrees in monocytes and/or DCs, whereas 53 miRNAs remained undetected, irrespective of cell type or stimulus (Additional file 3, Table S1). Intriguingly, 27 miRNAs appeared to be differentially expressed between monocytes, DCs and stimulated DCs (Table 1). These 27 miRNAs were selected for additional profiling of multiple donors as described below.

**Table 1:** MicroRNAs rescreened after primary screen in monocytes and dendritic cells, and their differences in expression level between cell types

MicroRNA in assay	Mature miRNA Sequence	Current genomic entries in miRbase matching mature miRNA	Up in DC	Up in DC subset	Up in monocytes	No difference
*hsa*-let-7e	UGAGGUAGGAGGUUGUAUAGU	same	X			

*hsa*-miR-15b	UAGCAGCACAUCAUGGUUUACA	same			X	

*hsa*-miR-16	UAGCAGCACGUAAAUAUUGGCG	hsa-miR-16-1, hsa-miR-16-2			X	

*hsa*-miR-27a	UUCACAGUGGCUAAGUUCCGCC	same				X

*hsa-*miR-27b	UUCACAGUGGCUAAGUUCUG	same				X

*hsa*-miR-34a	UGGCAGUGUCUUAGCUGGUUGU	same	X			

*hsa*-miR-99a	AACCCGUAGAUCCGAUCUUGUG	same	X			

*hsa*-miR-100	AACCCGUAGAUCCGAACUUGUG	same	X			

*hsa*-miR-125a	UCCCUGAGACCCUUUAACCUGUG	same	X			

*hsa*-miR-125b	UCCCUGAGACCCUAACUUGUGA	hsa-miR-125b-1, hsa-miR-125b-2	X			

*hsa*-miR-126	UCGUACCGUGAGUAAUAAUGC	same				X

*hsa*-miR-130a	CAGUGCAAUGUUAAAAGGGC	same			X	

*hsa*-miR-132	UAACAGUCUACAGCCAUGGUCG	same				X

*hsa*-miR-135a	UAUGGCUUUUUAUUCCUAUGUGA	hsa-miR-135a-1, hsa-miR-135a-2	X			

*hsa*-miR-137	UAUUGCUUAAGAAUACGCGUAG	same	X			

*hsa*-miR-140	AGUGGUUUUACCCUAUGGUAG	same				X

*hsa*-miR-146	UGAGAACUGAAUUCCAUGGGUU	hsa-miR-146a		X		

*hsa*-miR-150	UCUCCCAACCCUUGUACCAGUG	same			X	

*hsa*-miR-155	UUAAUGCUAAUCGUGAUAGGGG	same		X		

*hsa*-miR-186	CAAAGAAUUCUCCUUUUGGGCUU	same				X

*hsa*-miR-199b	CCCAGUGUUUAGACUAUCUGUUC	same			X	

*hsa*-miR-199-s	CCCAGUGUUCAGACUACCUGUU	hsa-miR-199a-1, hsa-miR-199a-2			X	

*hsa*-miR-210	CUGUGCGUGUGACAGCGGCUG	same		X		

### Identification of miRNAs differentially regulated during monocyte-derived DC activation

**Figure 1:**
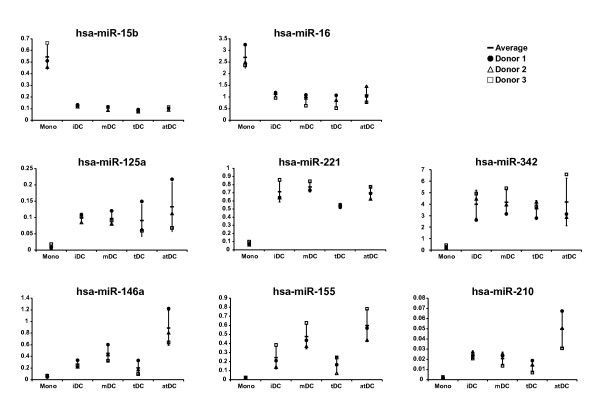
**Expression profiles of various miRNAs that are differentially expressed in monocytes and DCs**. The upper two charts represent miRNAs over-expressed in monocytes, the middle row of charts represent 3 of the miRNAs over-expressed in DCs, and the lower row of charts represent the 3 miRNAs that are differentially expressed among subsets of DCs. On the Y-axis relative expression is depicted, which is normalized to the expression of *hsa*-let-7a.

To validate the expression of the identified 27 miRNAs, their expression was further examined in monocytes and DCs of 3 different donors (Additional file 2, Figure S2). Statistical analysis revealed that 18 miRNAs were truly differentially expressed (gene-by-gene ANOVA, p < 0.05; Table 2). Six miRNAs were up-regulated in monocytes versus DCs, and 10 were up-regulated in DCs versus monocytes. Eight miRNAs were not differentially expressed and had a high degree of variation in expression levels (data not shown). However, *hsa-*miR-15b and *hsa-*miR-16 showed remarkably similar expression profiles and are expressed at higher levels in monocytes (Figure 1, upper row of charts; Pearson correlation = 0.99). Hsa-miR-125a, *hsa*-miR-221 and *hsa*-miR-342 are examples of miRNAs that are up-regulated in the DC populations relative to monocytes (Figure 1, middle row). Furthermore, both *hsa-*miR-146a and *hsa-*miR-155 were up-regulated during differentiation, and were slightly up-regulated in mDCs and atDCs (Figure 1, two left charts in lower row; gene-by-gene ANOVA, p < 0.05, Tukey's post-hoc test; Pearson correlation = 0.90), whereas *hsa-*miR-210 was primarily up-regulated in atDCs (Figure 1, right chart of lower row). Data generated from these analyses indicate that many of the selected miRNAs are indeed expressed in monocytes and DCs, and that 3 of these miRNAs are differentially regulated during DC development and maturation.

**Table 2:** Assignment of p-values for expressed miRNAs in all cell types (monocytes and various DC subsets) and DC subsets alone after ANOVA

	Posterior p-values*	
miRNA gene	All cell types	DC subsets

hsa-let-7e	1.9E-05	4.2E-01

hsa-miR-15b	1.2E-04	6.8E-01

hsa-miR-16	2.1E-02	7.8E-01

hsa-miR-27a	3.0E-01	2.5E-01

hsa-miR-27b	8.2E-01	8.2E-01

hsa-miR-34a	3.0E-05	9.6E-01

hsa-miR-99a	2.2E-06	7.2E-01

hsa-miR-100	3.7E-06	7.3E-01

hsa-miR-125a	1.3E-04	8.6E-01

hsa-miR-125b	1.6E-05	7.2E-01

hsa-miR-126	6.1E-01	7.7E-01

hsa-miR-130a	1.6E-03	4.4E-01

**hsa-miR-132	4.0E-02	9.8E-01

hsa-miR-135a	7.1E-10	9.1E-02

hsa-miR-137	3.2E-07	2.1E-01

hsa-miR-140	8.2E-01	7.1E-01

hsa-miR-146a	9.6E-05	1.3E-02

***hsa-miR-150	9.9E-02	7.1E-01

hsa-miR-155	4.7E-06	2.5E-02

hsa-miR-186	7.2E-01	6.9E-01

hsa-miR-199b	5.2E-05	7.1E-01

hsa-miR-199s	8.4E-03	4.4E-01

hsa-miR-210	1.3E-05	4.0E-02

hsa-miR-221	5.6E-06	7.4E-01

hsa-miR-326	7.4E-01	9.7E-01

hsa-miR-340	8.6E-01	7.9E-01

hsa-miR-342	6.1E-06	9.9E-01

*) Posterior p-values < 0.05 indicate differential expression among subsets.

**) Considered not expressed differentially after visual inspection of gene expression data.

***) Considered expressed differentially after visual inspection of the data.

### Identification of TFBSs in promoters of differentially expressed miRNAs in monocytes and DCs

**Figure 2:**
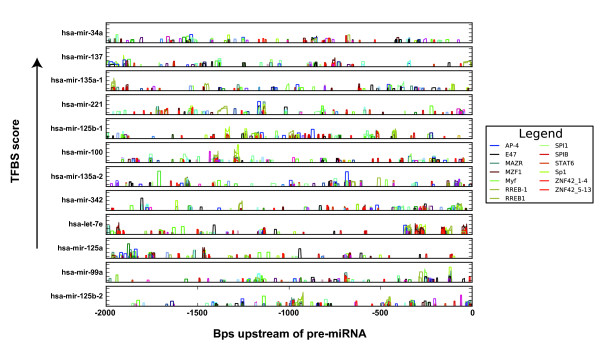
**Representation of TFBSs in promoter regions of miRNAs up-regulated in DCs**. Depicted are the TFBSs at a motif instance score threshold of at least 6 in the miRNA promoter regions. The scale of the y-axis ranges from 5 to 15 for each subgraph. The legend shows only those TFBSs that are present in all promoter sequences.

**Figure 3:**
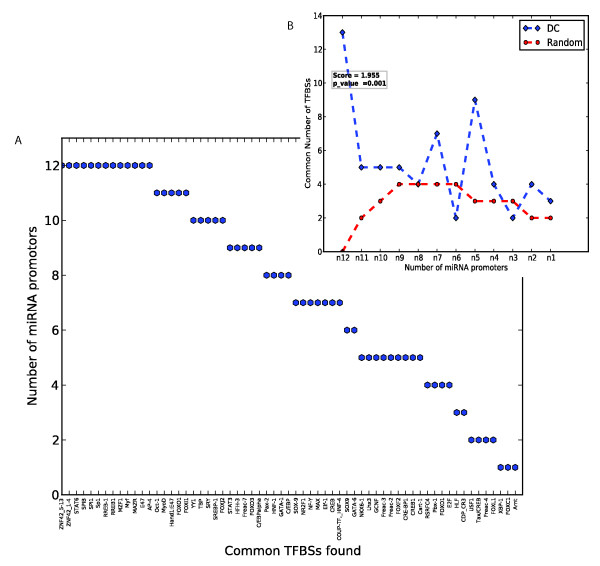
**TFBSs shared among the promoter regions of miRNAs up-regulated in DCs**. (A) Shown on the x-axis are the high-scoring TFBSs (i.e. of instance score ≥ 6) that occur at least once in the 2 kb promoters of the miRNA up-regulated in DCs. The y-axis shows the number of promoters that have the TFBS at least once at this threshold. (B) The distribution of the number of common TFBS hits per number of common miRNA promoters as in A, for DCs and random sets of miRNA promoters. The values for random are the median values from 1000 randomly chosen sets of 12 miRNA promoters. The score is the ratio of the sum of all TFBS occurrences across all promoters for the DC set relative to that of the random set. The p-value is the fraction of cases wherein this sum for random sets of miRNA promoters is greater than or equal to that of the DC set.

In order to link the miRNA expression profiles to a specific TFBS (or groups of TFBSs) of the myeloid cell types that were analyzed, sequences 2 kb upstream of the mapped pre-miRNAs were chosen for analysis. It should be noted that it is expected that a majority of miRNAs contain their own promoters [21], and that in some cases, different miRNA genes (such as *hsa*-miR-16-1 and *hsa*-miR-16-2) in the genome give rise to an identical mature miRNA (such as *hsa*-miR-16). Therefore 31 promoters could be assigned to 27 miRNAs that were profiled in detail (Table 1). Likewise, 167 promoters could be assigned to the total of 157 miRNAs analyzed. Analysis of the 31 promoters revealed that 3 TFBS motifs (Elk-1, RREB-1 and SPIB) were over-represented using the Clover criteria (p < 0.05) among all 8 promoter sequences of miRNA genes that are up-regulated in monocytes, and to have at least 1 high-scoring TFBS (instance score ≥ 6) in each promoter. Selecting TFBSs using these same criteria (over-represented motifs and at least 1 high-scoring TFBS per promoter) among the 12 promoters of the miRNAs that are up-regulated in DCs relative to monocytes, reveals that they have 13 TFBSs in common (Figure 2, Table 3, Additional file 4, Table S2). Furthermore, 4 TFBSs are over-represented in the promoters in all of the 8 miRNAs that did not show any appreciable difference in expression levels (Table 3). The complete set of high-scoring TFBSs found in the promoters of the miRNAs that are up-regulated in DCs and monocytes are summarized in Figure 3 and Additional file 5, Figure S3 respectively.

**Table 3:** TFBSs that are present in all miRNA promoters in a particular (group of) cell type(s)

Cell types	Overrepresented TFBS
Monocytes	Elk-1, RREB-1, SPIB

DCs*	AP-4, E47, MAZR, Myf, MZF1, RREB1, RREB-1, Sp1, SPI1, SPIB, STAT6, ZNF42_1-4, ZNF42_5-13

Subset of DCs**	AP-1, ARP-1, FOXI1, myogenin/NF-1, Ncx, Oct-1, OCT-x, Sp1, TCF11, ZNF42_1-4

All cells***	Oct-1, RP58, SPIB, USF

*) Includes monocyte-derived iDCs, maturing DCs, tolerogenic DCs and activated tolerogenic DCs

**) Includes maturing DCs and/or activated tolerogenic DCs

***) Concerns all cells investigated, and miRNAs did not show appreciable differences in expression level

Clover detects motifs that are over-represented relative to their expected frequencies and calculates p-values based on a comparison with all promoters in the genome. Nevertheless such predictions can potentially give rise to false positives, as even random sets of miRNA promoters contain some over-represented motifs (Figure 3B). We therefore also examined the over-representation of motifs in the promoters of DC and monocyte miRNAs relative to those of random sets of miRNAs, selected from the total set of 167 miRNA promoters. The 12 miRNAs that are over-expressed in DCs share more TFBS motifs than do random sets of 12 miRNA promoters, specifically with respect to motifs that are shared by many of the promoters (Figure 3B). When quantifying the number of shared motifs as the sum of all occurrences of all Clover detected motifs, across all promoters, we observe an enrichment of 1.96 relative to randomly selected miRNA promoters (p = 0.001). Likewise, using random sets of 8 miRNA promoters, we observed that the 8 miRNAs that are over-expressed in monocytes have a factor of 1.58 enrichment of TFBS motifs (p = 0.046; Additional file 5, Figure S3B). The p-value here was estimated as the number of times, out of 1000, when a randomly chosen set of miRNA promoters had at least an equal number of motifs as did the test set of sequences. Given the significant over-representation of different sets of TFBSs in the different sets of miRNAs, it appears that miRNAs contain multiple, different TFBSs in their promoters that specify their expression in certain cell types. This is in agreement with other studies that show that transcription factors often work in combinations [25,26].

### Evolutionary conservation and distribution of TFBSs in the promoter sequences of myeloid miRNAs

**Figure 4:**
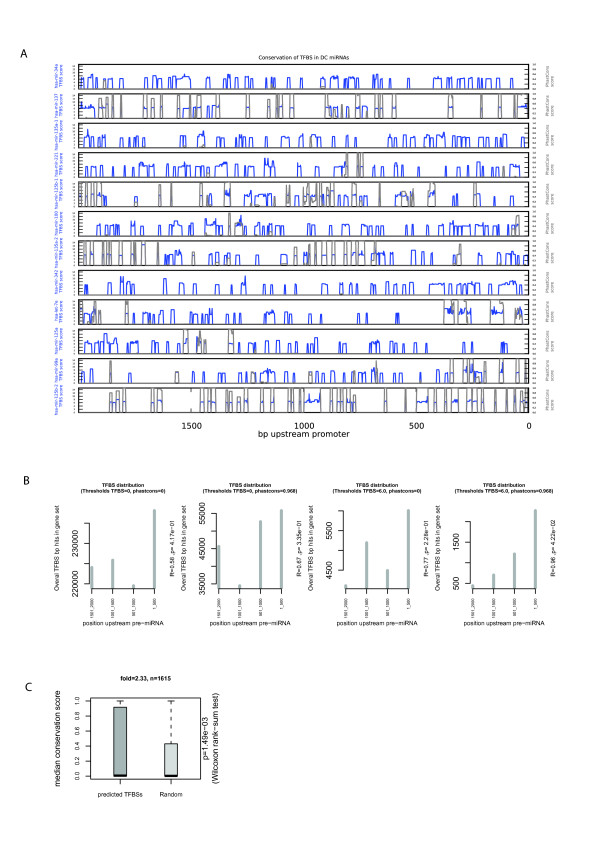
**Distribution and evolutionary conservation of TFBSs in promoter regions of DC-expressed miRNAs**. (A) The TFBS motif scores (in blue, y-axis range from 0 to 15 per subgraph) as calculated using Clover and the extent of evolutionary conservation as PhastCons score (in grey, y-axis on the right). (B) Distribution pattern of TFBSs, selected based on various motif score and evolutionary score thresholds, in the promoter region of miRNAs up-regulated in DCs. (C) Comparison of the median conservation scores of the bases in the predicted TFBSs in the promoters of miRNAs that are over-expressed in DCs, with those of randomly chosen sequences of the same length from the same promoters. Boxplots represent the median and interquartile range of the median PhastCons conservation scores. The fold shown is the ratio of the median of the DC conservation scores and the median of the conservation scores of the random sets (n = number of TFBS instances concerned).

Assuming that regulation of miRNA expression is conserved among mammals, we used the PhastCons evolutionary conservation track that is based on nucleotide conservation among placental mammals (http://hgdownload.cse.ucsc.edu/goldenPath/hg18/phastCons44way/) to quantify evolutionary conservation of the predicted TFBSs. We compared both the motif instance scores and raw scores of TFBSs, as derived from Clover, with the nucleotide conservation score of the TFBSs. Applied to the 2 kb upstream sequences of the pre-miRNAs that are up-regulated in DCs, a small but significant correlation of 0.12 (p = 6.17 × 10^-7^) was observed between the conservation scores and the TFBS instance scores. Similar results were obtained using the raw score (r = 0.22, p = 8.99 × 10^-2^). An overlay of TFBS instance scores and conservation scores for the DC miRNA promoters is depicted in Figure 4A. The positive but weak correlation between TFBS motif scores and conservation scores suggests that they are largely independent measures and as such, combination of the two as a filtration measure would be non-redundant. When filtering predicted sites based both on their conservation and on their TFBS instance scores, TFBSs tend to cluster in a region 500 bp just upstream of the putative TSS of miRNA genes that are over-expressed in DCs (Figure 4B). This result corroborates other findings indicating that TFBSs tend to cluster near the transcription start site (TSS) of genes [51-54]. In addition, it suggests that combining the motif matching score with the extent of evolutionary conservation of a TFBS would likely reveal TFs biologically relevant to the identified set of miRNA promoters.

**Figure 5:**
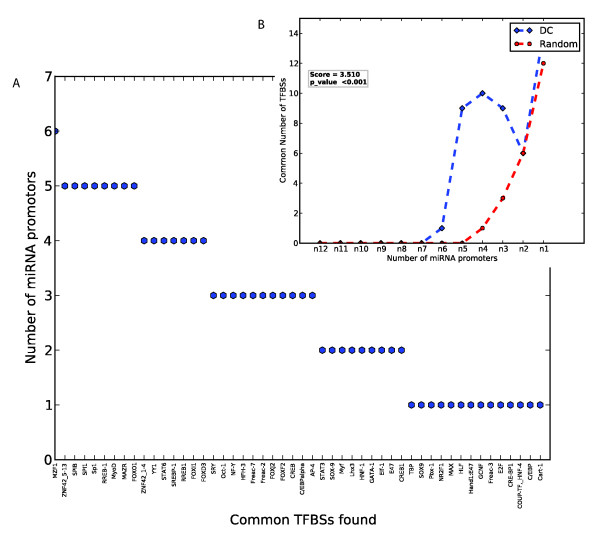
**(A) Degree to which conserved TFBSs in promoter regions of miRNAs up-regulated in DCs are shared among their promoters**. (B) The distribution of the number of common TFBS hits per number of common miRNA promoters as in A, for test and random sets of miRNA promoters. (A) Degree to which conserved TFBSs in promoter regions of miRNAs up-regulated in DCs are shared among their promoters. Shown on the x-axis are high-scoring TFBSs filtered at a motif instance score threshold of at least 6 and at a 10^th ^percentile evolutionary conservation score that occur at least once in the promoters of miRNAs up-regulated in DCs. The y-axis shows the number of promoters that have the TFBS at least once at these thresholds. (B) The distribution of the number of common TFBS hits per number of common miRNA promoters as in A, for test and random sets of miRNA promoters. The values for random are the median values from 1000 random sets of miRNA promoters of the same size and length as those in the DC set. The score is the ratio of the sum of all occurrences of conserved TFBS for the DC set relative to that of the random sets. The p-value is estimated from the number of instances wherein this sum for 1000 random sets of miRNA promoters is greater than or equal to that of the DC set.

In further support of the conservation of the binding motifs that are over-represented in the promoters of the DC-expressed miRNAs, we created a set of random sequence blocks of the same length-distribution as the predicted TFBSs from the same promoters as the test set. From these two data sets, we observed that the predicted TFBSs are significantly (p = 1.49 × 10^-3^, Wilcoxon rank sum test) more conserved than randomly chosen sequences. The conservation score assigned to a TFBS is the median of the PhastCons scores of the bases in that TFBS segment. We observed that the median of the conservation scores of the test TFBSs is more than 2 fold higher than that of the random sequences (Figure 4C). The complete set of conserved high-scoring TFBSs found in the promoters of the miRNAs up-regulated in DCs and monocytes are summarized in Figure 5 and Additional file 6, Figure S4 respectively. Furthermore, we observed at the threshold of 10^th ^percentile of median TFBS conservation, that the miRNAs that are over-expressed in DCs share more (fold = 3.51, p < 0.001) sites for TF motifs than did 1000 random sets of promoters of equal size, length and at the same conservation threshold (Figure 5B). A similar result (fold = 3.48, p = 0.003) was obtained for the miRNAs that were over-expressed in monocytes (Additional file 6, Figure S4B). These results show that evolutionary conserved TFBSs are more common in the promoters of miRNAs over-expressed in DCs and monocytes than in randomly chosen miRNA promoters.

### Integrating the motif-matching and evolutionary conservation scores of TFBSs and further validation of cognate TFs in microarray data from DCs

Without taking evolutionary information into account, 13 TFBSs were found to occur at least once in all the promoters of miRNAs preferentially expressed in DCs (Figure 2). Filtering TFBSs at 10^th ^percentile threshold of the TFBS conservation scores not surprisingly identifies TFBSs that are common to far less miRNAs promoters (Figure 5). Among these, the TFBS of MZF1 was the most conserved at the thresholds used. To validate the expression of the cognate transcription factors whose binding site was identified, a transcriptome was generated of DCs from one donor using microarrays. The resulting data were screened for the presence or absence of transcription factors (TFs) whose binding sites were predicted in promoters of miRNAs up-regulated in DCs. For 10 out of the 17 TFBSs that are over-represented among the DC promoters (Additional file 4, Table S2B) and that contained at least one TFBS with an instance score of at least 6.0 and a conservation threshold score of 0.968 (i.e. the 10^th ^percentile of the median conservation scores of all TFBSs predicted in the set of miRNA promoters) as shown in Figure 5, the mRNA encoding their cognate TF was expressed in DCs. Expression of this 58.8% of the predicted over-represented TFs is slightly higher than that for all the TFs that were examined with the microarrays (54% expressed, 46% not expressed, Additional file 7, Table S3). Nevertheless, we found a high correlation (r = 0.91, p = 1.17 × 10^-2^) when the number of motifs of expressed TFs was correlated with the number of miRNA promoters in which the motifs of these TFs were detected (Additional file 8, Figure S5A). An insignificant correlation (r = -0.72, p = 4.88 × 10^-1^) was observed when a random set of miRNA promoters was used (Additional file 8, Figure S5B).

Gene Ontology of the expressed TFs highlighted immune-related processes like "interspecies interaction between organisms" (p = 4.49 × 10^-5^) and "regulation of T-helper 2 type immune response" (p = 1.19 × 10^-2^, Additional file 9, Table S4A). This is in line with the expected functions of DCs in immune response. Meanwhile, Gene Ontology of the TFs that were not expressed highlighted development-related processes like "cell development" (p = 1.88 × 10^-3^) and "cell fate commitment" (p = 7.54 × 10^-3^, Additional file 9, Table S4B), with little relevance to immune-related processes. Taken together, these data provide evidence that many expressed miRNAs in DCs have evolutionarily conserved TFBSs that may be relevant to DC biology in their promoters.

### Target prediction and Gene Ontology analysis of microRNAs enriched in monocytes and DCs

To extend the regulatory pathways in which the miRNAs participate downstream, we predicted miRNA targets, using TargetScanHuman version 5.1 [15] and PicTar [46]. Both datasets were based on conservation in mammals. We obtained the precompiled dataset from their official websites. At a TargetScan context score threshold of at most -0.4, we obtained 421 distinct target genes (Additional file 10, Table S5; counting in RefSeq DNA IDs) for the miRNAs that are over-expressed in DCs. Meanwhile at a PicTar score threshold of at least 0.4, we obtained 2300 distinct target genes (Additional file 11, Table S6) for the miRNAs over-expressed in DCs. Likewise, we predicted 282 (Additional file 12, Table S7) and 1591 (Additional file 13, Table S8) distinct genes using TargetScan and PicTar respectively, to be targets for the miRNAs that are over-expressed in monocytes. For both DC-over-expressed miRNAs and monocyte-over-expressed miRNAs, the overlap of targets predicted by both TargetScan and PicTar is less than 50% of their respective total predictions (Additional file 14, Figure S6) suggesting that we still need to examine their independent predictions. Interestingly, only 7 identical targets were predicted for both monocyte-over-expressed miRNAs and DC-over-expressed miRNAs at the TargetScan threshold of ≤-0.4 and PicTar threshold of ≥0.4. The numbers of target genes predicted are summarized in Additional file 14, Figure S6.

The number of target genes predicted using TargetScan and PicTar are very large even at the stringent thresholds used. Furthermore, Gene Ontology (GO) analyses of the gene sets did not yield conclusive results. To increase the relevance of the predicted targets to DCs we further filtered them based on their differential expression in DCs relative to monocytes during DC differentiation using previously published data by Lehtonen *et al *(2007) [47]. At a gene expression fold change threshold of 2, we identified 34 unique genes corresponding to the DC-associated targets predicted using PicTar to be down-regulated at all time points in DCs relative to monocytes (Additional file 15, Table S9A). Amongst this filtered set of genes is *IL10*, which interestingly is involved in the process of "negative regulation of myeloid dendritic cell activation" (p = 2.64 × 10^-2^, from GO analysis of the 34 down-regulated genes). Correspondingly, 10 unique genes were associated with the down-regulated genes of the TargetScan-predicted targets of miRNAs that were over-expressed in DCs relative to monocytes (Additional file 15, Table S9B). In total, 6 of these down-regulated genes (*SIK1, PELI2, MYLIP, RNF138, CCNG2 *and *FOXO1; *in order of increasing down-regulation of their RNA transcripts at time point 24 hr) were also predicted using PicTar (Additional file 15, Table S9). Of these, *SIK1, PELI2 *and one transcript of *CCNG2 *were increasingly down-regulated in DCs relative to monocytes in the time course of DC differentiation from monocytes; suggesting their involvement in mechanisms of DC differentiation.

In addition to the DC dataset, we also examined the expression of the predicted target genes of the miRNAs that are over-expressed in monocytes relative to DCs. We found 35 unique genes associated with the PicTar-predicted targets of miRNAs over-expressed in monocytes relative to DCs that were down-regulated in monocytes relative to DCs (Additional file 16, Table S10A). Correspondingly, transcripts of 9 unique genes associated with the TargetScan-predicted targets of miRNA over-expressed in monocytes relative to DCs were also down-regulated at all time points in monocytes relative to DCs (Table S10B). Among these is *DICER1 *which is involved in RNA interference and is up-regulated in DCs. Furthermore, there was an overlap of 2 genes (*ACVR2A *and *MAP3K4*) between the PicTar and TargetScan target gene sets (Additional file 14, Figure S6 and Additional file 17, Table S11). One transcript of *ACVR2A *was increasingly down-regulated (Additional file 16, Table S10). Moreover, there was no overlap in expressed genes that were targets to both DC- and monocyte-over-expressed miRNAs, suggesting congruency in our data. These results suggest that miRNA target genes can be either up-regulated or down-regulated in myeloid cells to regulate differentiation of the myeloid cells.

## Discussion

As little is known about the expression and regulation of miRNAs in monocytes, DCs and their stimulated progeny, part of the miRNA transcriptome of these cells was generated and analyzed. Out of 157 miRNAs profiled, 104 appeared to be expressed in myeloid cells to a varying degree. Since the database miRBase 13.0 (March 2009) reveals the existence of at least 706 human miRNAs, it is conceivable that a much higher number of miRNAs is expressed in human monocytes and DCs. Six miRNAs appear up-regulated in monocytes, 10 are up-regulated in DCs and 3 are differentially expressed among different DC populations in response to LPS. Also, some miRNAs showed similar changes in expression levels among DC subsets.

Amongst the 10 miRNAs that were over-expressed in DCs as compared to the monocytes from which they were derived, *hsa*-miR-34a, *hsa-*miR-125a, *hsa*-miR-342 and *hsa*-let-7e have been shown to be up-regulated in the course of DC differentiation from monocytes in culture [55,56]. The up-regulation of *hsa*-miR-342 in monocyte-derived DCs as compared to monocytes is likely the result of culture conditions, as *hsa*-miR-342 is expressed at a much lower level in freshly isolated blood-derived myeloid DCs than in DCs generated *in vitro *(data not shown). Of the remaining miRNAs, 6 show an upward trend in expression level in blood-derived DCs, but the levels in cultured DCs are higher. Intriguingly, *hsa*-miR-146a and *hsa*-miR-155 appear to respond to LPS, but much less so than in monocytes and macrophages [7,8]. Recently, it was demonstrated that LPS-mediated activation of protein kinase Akt1 results in up-regulation of miRNA let-7e in primary macrophages, while at the same time repressing miR-155 expression [57]. Our data show that both *hsa*-let-7e and *hsa*-miR-155 are up-regulated in DCs compared to monocytes, but that only *hsa*-miR-155 is slightly up-regulated by LPS. Taken together, these data imply that intrinsic differences between DCs and macrophages exist in response to the TLR4 ligand LPS.

With regard to the targets of the miRNAs screened herein, recent literature indicates that inhibition of *hsa*-miR-34a or addition of one of its target genes, JAG1, have been observed to functionally stall the differentiation of monocyte-derived dendritic cells [55]. This supports our identification of *hsa*-miR-34a as an over-expressed miRNA in DCs relative to monocytes and JAG1 as its target gene (NM_000214 in Additional file 11, Table S6). In addition, we provide a list of predicted DC targets that are down-regulated in expression in DCs relative to monocytes (Additional file 15, Table S9). In this list, one of the targets of the DC-expressed miRNA, *hsa*-let-7e, is IL10, which is involved in negative regulation of myeloid dendritic cell activation. Taken together, these data highlight miRNAs, and their target genes, that can potentially modulate DC differentiation.

Extensive miRNA promoter analysis revealed that 13 TFBSs are over-represented and commonly shared by the 12 promoter sequences of the 10 miRNAs that are up-regulated in DCs. These include *signal transducer and activator of transcription 6 *(STAT6), which is known to be involved in IL4 signaling and DC differentiation and maturation [58], *spleen focus forming virus (SFFV) proviral integration oncogene spi1 *(SPI1, also known as PU.1), which is indispensable for normal myeloid and lymphoid development [59] and *specificity protein 1 *(SP1), which is involved in the expression of *dendritic cell-specific ICAM-3 grabbing non-integrin *(DC-SIGN) [60].

Although the TSSs in these promoter regions are not known, TFBSs did cluster, especially when taking evolutionary conservation into account, within the 500 bp upstream region of the annotated pre-miRNAs, coinciding with the region in miRNA promoters at which TSS have been discovered experimentally [19,61]. Amongst the TFBSs predicted in the DC miRNAs, the sites for *myeloid zinc finger *(MZF1) is best conserved of all, even though the role of its cognate TF in DCs remains elusive. Nevertheless, MZF1 is thought to be a bi-functional transcriptional regulator, repressing transcription in non-hematopoietic cells, activating transcription in cells of hematopoietic origins and controlling cell proliferation and tumorigenesis [62,63]. In addition, one of the conserved TFBSs for which the cognate TF was not expressed in monocyte-derived DCs appears to be *Spi-B transcription factor *(SPIB), which has been implicated in plasmacytoid DC (pDC) development, a non-myeloid cell type [64,65]. It should be noted, however, that many of the miRNAs expressed in myeloid monocyte-derived DCs are also expressed in pDCs (data not shown).

When taking all predicted and well-conserved TFBSs in the promoters of miRNAs that are up-regulated in DCs into account, the number of TFs expressed increases with the degree at which their TFBS motifs are shared between the promoter sequences. Gene Ontology analysis indicates that the type of expressed TFs enriched in these data are relevant to the immune system process and are in line with the known function of DCs, whereas those that were not expressed are relevant to cell development (Additional file 9, Table S4). Importantly, libraries of TFBS motifs used do not represent all possible TFBSs and as such not all possible TFBSs for expressed miRNAs have been identified in this study. Moreover, for consistency, we have used TFBS motifs, and not TFs, in making comparisons of TFBSs because the databases used have redundant motif names for the same TFs. It should be noted that predicted target genes were not uniformly down-regulated, as we found evidence in DCs whereby predicted target genes were up-regulated in the cells or were also targets for the miRNAs that were over-expressed in monocytes. This may be due to limitations of the target prediction algorithms, or the targets might still be down-regulated at the protein level. Nevertheless, none of the down-regulated DC-miRNA targets was a predicted target for the monocyte-miRNAs. Furthermore, there is evidence that in a minority of cases, target genes are actually up-regulated by miRNAs [66,67]. The up-regulation of *DICER1 *in DCs relative to monocytes is of special interest, as it is known that innate immune signaling is tightly controlled by miRNAs [7,8]. Furthermore, there is also evidence that one type of DC, the Langerhans cell, requires proper functioning of DICER1 to induce CD4 T cell function [68]. Together with the fact that there are more up-regulated miRNAs in DCs than monocytes in our data set, it is tempting to speculate that *DICER1 *up-regulation is required for proper DC function.

## Conclusions

The data provide evidence that, among the many expressed miRNAs in DCs, evolutionarily conserved TFBSs relevant to DC biology are present in their promoters. Furthermore, the identified miRNAs, their associated TFs and predicted target genes could help improve our understanding of the molecular pathways that underpin DC differentiation and maturation.

## Abbreviations

hsa: Homo sapiens; miRNA: microRNA; DC: dendritic cell; iDC: immature DC; mDC: mature DC; tDC: tolerogenic DC; atDC: activated tolerogenic DC; pDC: plasmacytoid DC; TFBS: transcription factor binidng sites; TF: transcription factor; TSS: transcription start site; RISC: RNA-induced signaling complex; TLR: toll-like receptor; TNF: tumor necrosis factor; FACS: fluorescence-activated cell sorting; PCR: polymerase chain reaction; HMM: hidden markov modeling; GO: Gene Ontology; ANOVA: analysis of variance

## Authors' contributions

BJHJ conceived the study, carried out the miRNA profiling and mRNA profiling, performed statistical analysis on the data, participated in the bioinformatic analysis, carried out microarray experiments and drafted the manuscript. IES conceived the study, participated in the statistical analysis of the data, performed the bioinformatic analysis, and drafted the manuscript. DET cultured cells and analyzed cellular phenotypes. MAvHK participated in cell culture, sample preparation and miRNA profiling. JHJ participated in the design of the study and provided miRNA assays. MAH participated in the design and coordination of the study. GJA participated in the design and coordination of the study. All authors read and approved the manuscript.

## Authors' information

BJHJ and IES contributed equally to this manuscipt. BJHJ is now at Lead Pharma BV, Nijmegen, the Netherlands.

## Supplementary Material

Additional file 1 **Figure S1**. Setup of culture and quality of monocyte-derived DCs. (A) Culture setup and harvest schedule for RNA isolations. (B) Purity of monocytes (upper histogram) and the expression of maturation markers CD80 and CD86 on the different DC populations (C) Mixed lymphocyte reaction with the various DC populations; ratio of DCs vs. PBLs is indicated on the x-axis. (D) Production of TNFα and IL8 by the different DC populations, as determined by ELISA. The white and black bars each represent data from two different donors. Click here for file

Additional file 2 **Figure S2**. Schematic overview of the miRNA expression screen in monocytes and DCs. Click here for file

Additional file 3 **Table S1**. MicroRNAs for which assays have been developed by Applied Biosystems, and of which expression levels were determined in monocytes and dendritic cells. Of a total of 157 miRNAs, 104 were expressed in DC and/or monocytes, whereas 53 were not detected in either cell type. Click here for file

Additional file 4 **Table S2**. Overview of over-represented TFBS motifs in promoter seqeunces of miRNAs that are upregulated in monocytes or DCs. Click here for file

Additional file 5 **Figure S3**. (A) Commonality of TFBSs in promoter regions of monocyte-expressed miRNAs. Shown on the x-axis are the high-scoring TFBSs (i.e. of instance score ≥ 6) that occur at least once in the 2 kb promoters of the miRNA up-regulated in monocytes. The y-axis shows the number of promoters that have the TFBS, at least once at this threshold. (B) The distribution of the number of common TFBS hits per number of common miRNA promoters as in A, for test and random sets of miRNA promoters. The values for random are the median values from 1000 random set of miRNA promoters of same size and length as those in the monocyte set. The score is the ratio of the sum of "the product of numbers on the x-axis and corresponding y-axis values" for the monocyte set relative to that of the random. The p-value is the fraction of cases wherein this sum for random sets of miRNA promoters is greater than or equal to that of the DC set. Click here for file

Additional file 6 **Figure S4**. (A) TFBSs shared among the promoter regions of miRNAs up-regulated in monocytes. Shown on the x-axis are high-scoring TFBSs filtered at a motif instance score threshold of atleast 6 and at a 10^th ^percentile evolutionary conservation score that occur at least once in the promoters of miRNAs up-regulated in DCs. The y-axis shows the number of promoters that have the TFBS at least once at these thresholds. (B) The distribution of the number of common TFBS hits per number of common miRNA promoters as in A, for test and random sets of miRNA promoters. The values for random are the median values from 1000 random set of miRNA promoters of same size and length as those in the monocyte set. The score is the ratio of the sum of all occurrences of all TFBS for the monocyte set relative to that of the random sets. The p-value is estimated from the number of instances wherein this sum of "the product of number of TFBSs and the number of miRNA promoters" of 1000 random sets of miRNA promoters is greater than or equal to that of the monocyte set. Click here for file

Additional file 7 **Table S3**. Library of TFBS motif for which the cognate TFs are expressed (presence = 1) or not expressed (absence = 1) in DCs. Click here for file

Additional file 8 **Figure S5**. Correlation of the number of expressed TFs with the number of promoters of miRNAs that are over-expressed in DCs. The TFs used are those of high-scoring TFBSs (i.e. of instance score > = 6) that are also highly conserved (10th percentile of PhastCons scores) and occur at least once in the 2 kb promoters of the miRNA up-regulated in DCs (left plot), and a random set of miRNAs (right plot). The original number of miRNA promoters that share at least one TFBS was 12 for both DCs and the random set of promoters (i.e the number of promoters of miRNAs that were over-expressed in DCs). Due to the high conservation threshold used, the maximum number of miRNA promoters that share at least one TFBS became smaller (6 for the test set and 3 for the random set). The correlations were calculated using the Pearson correlation as implemented in R. Click here for file

Additional file 9 **Table S4**. Gene ontology analysis of the expressed and not-expressed TFs of predicted binding sites in the promoters of miRNA. Click here for file

Additional file 10 **Table S5**. The 421 target genes that are predicted by TargetScan for the DC-over-expressed miRNAs. Click here for file

Additional file 11 **Table S6**. The 2300 target genes that are predicted by PicTar for the DC-over-expressed miRNAs. Click here for file

Additional file 12 **Table S7**. The 282 target genes that are predicted by TargetScan for the Monocyte-over-expressed miRNAs. Click here for file

Additional file 13 **Table S8**. The 1591 target genes that are predicted by PicTar for the Monocyte-over-expressed miRNAs. Click here for file

Additional file 14 **Figure S6**. Venn diagram showing the intersection of target genes. (A) Intersection of target genes of the miRNAs that are over-expressed in DCs, using PicTar (DC.pic), and TargetScan (DC.tar). (B) Similar to A, but for monocytes. (C) Intersections of the common targets found by PicTar and TargetScan for DC miRNAs (DC.pic.tar), the common targets for monocyte miRNAs (mono.pic.tar), and the dataset of Lehtonen et al. 2007 (Leh.) of genes that are regulated in DCs relative to monocytes during DC differentiation. (D-F) Intersection of common target genes from DC miRNAs, monocyte miRNAs, and genes that were up-regulated (upreg) or down-regulated (downreg), in DCs relative to monocytes in the data set of Lehtonen et al.2007. Figure D, E, F represent respectively the data sets at time points 3, 6 and 24 hr of DC differentiation from monocytes. (Differential gene relations (DC/monocyte) were selected at a fold change of at least 2. The PicTar score used > = 0.4, TargetScan context score < = -0.4. Comparisons were done at the level of RefSeq DNA ID and the elements in the intersections with the upreg and downreg datasets are provided in Table S11 with gene symbols attached. Click here for file

Additional file 15 **Table S9**. Target genes that are down-regulated in DCs relative to monocytes and that were predicted using PicTar or Targetscan, to be targets of miRNAs that are over-expressed in DCs relative to monocytes. Click here for file

Additional file 16 **Table S10**. Target genes that are down-regulated in monocytes relative to DCs and that were predicted using PicTar or Targetscan, to be targets of miRNAs that are over-expressed in monocytes relative to DCs. Click here for file

Additional file 17 **Table S11**. Elements in the Venn diagram intersection of Figure S6D to S6F. Click here for file
