# Supplementary figures and images for: MicroRNA genes preferentially expressed in dendritic cells contain sites for conserved transcription factor binding motifs in their promoters
Source: BMC Genomics. 2011 Jun 27;12:330. doi: 10.1186/1471-2164-12-330 (PMC3146452; doi:10.1186/1471-2164-12-330)

Figure S1

A

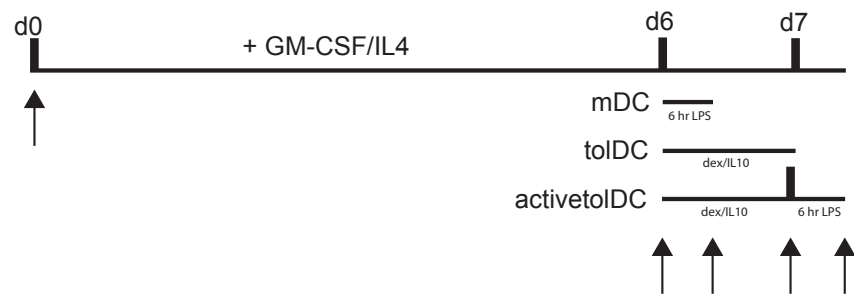

B

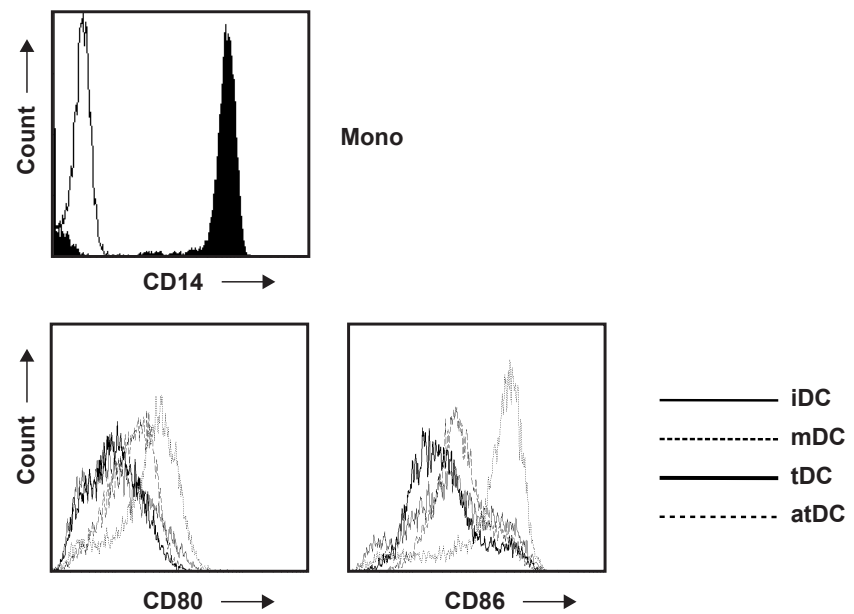

C

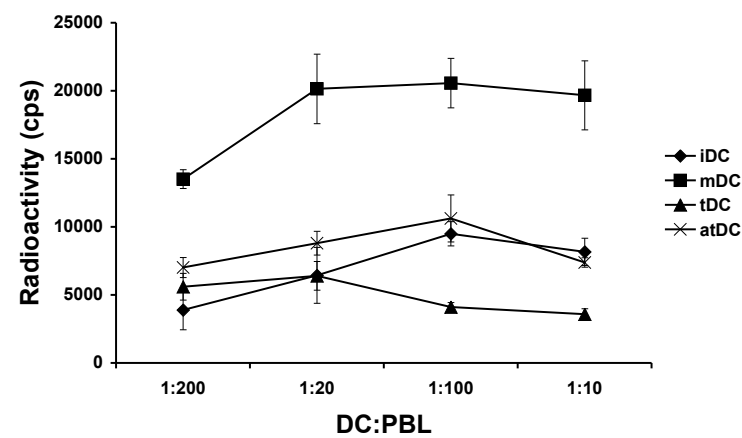

D

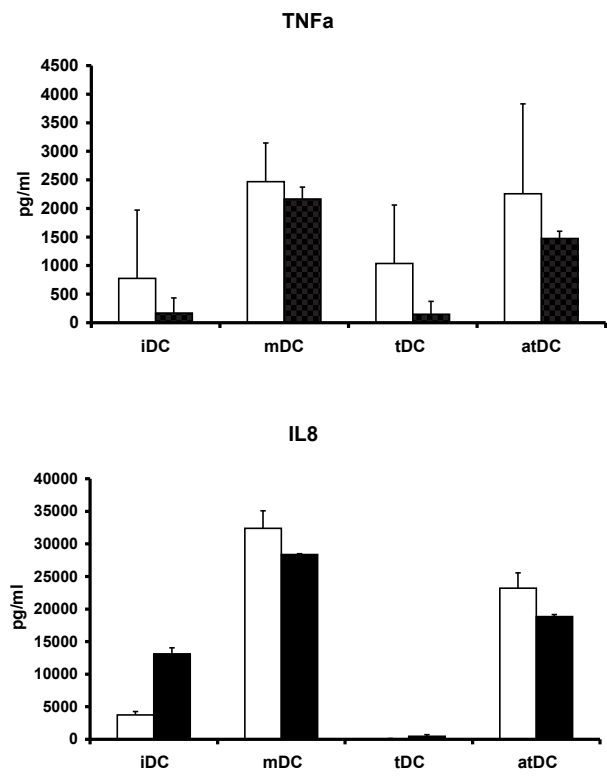

Supplement: Additional file 1 — Figure S1. Setup of culture and quality of monocyte-derived DCs. (A) Culture setup and harvest schedule for RNA isolations. (B) Purity of monocytes (upper histogram) and the expression of maturation markers CD80 and CD86 on the different DC populations (C) Mixed lymphocyte reaction with the various DC populations; ratio of DCs vs. PBLs is indicated on the x-axis. (D) Production of TNFα and IL8 by the different DC populations, as determined by ELISA. The white and black bars each represent data from two different donors. [file 1471-2164-12-330-S1.PDF]

Figure S2

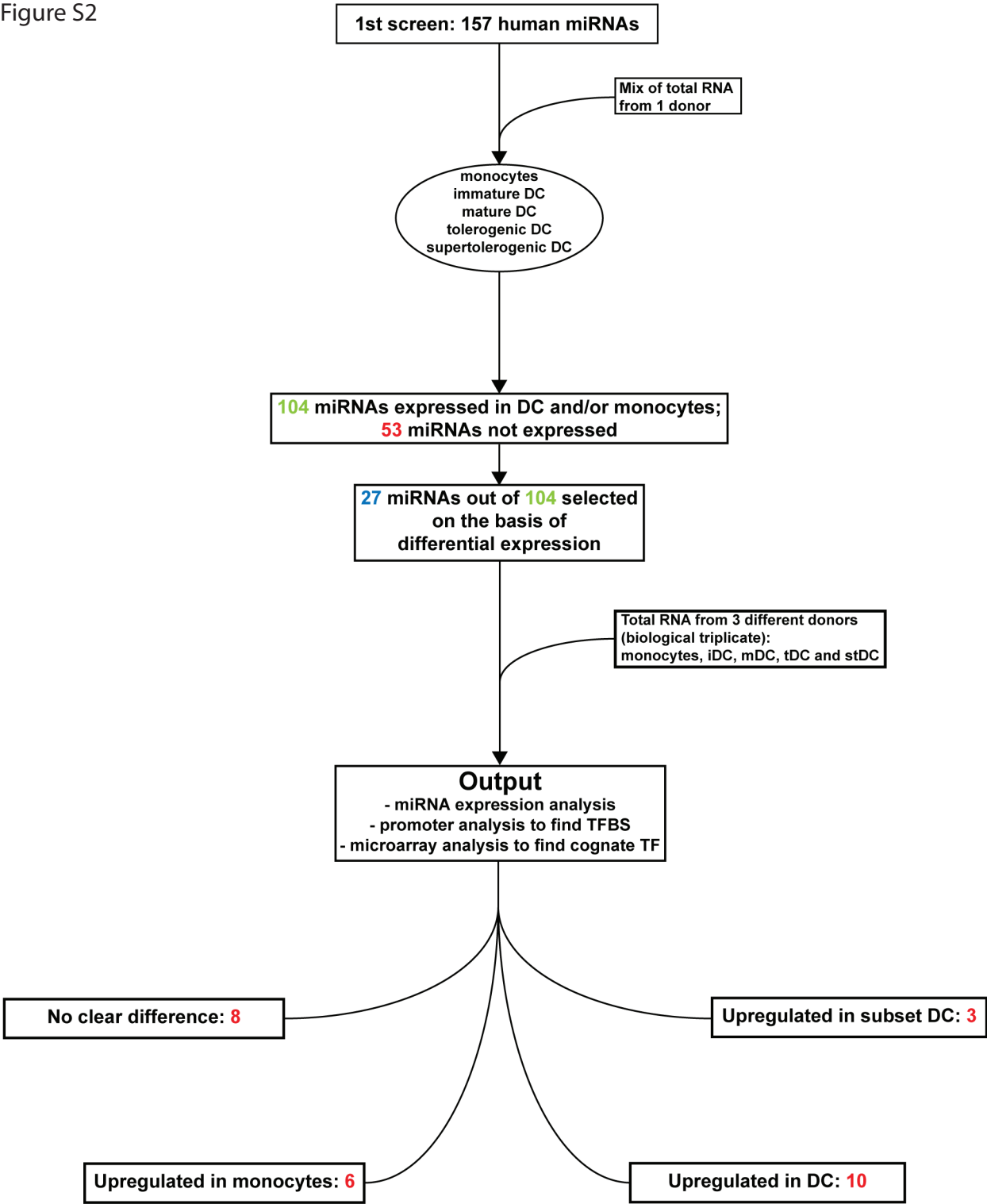

Supplement: Additional file 2 — Figure S2. Schematic overview of the miRNA expression screen in monocytes and DCs. [file 1471-2164-12-330-S2.PDF]

Figure S3

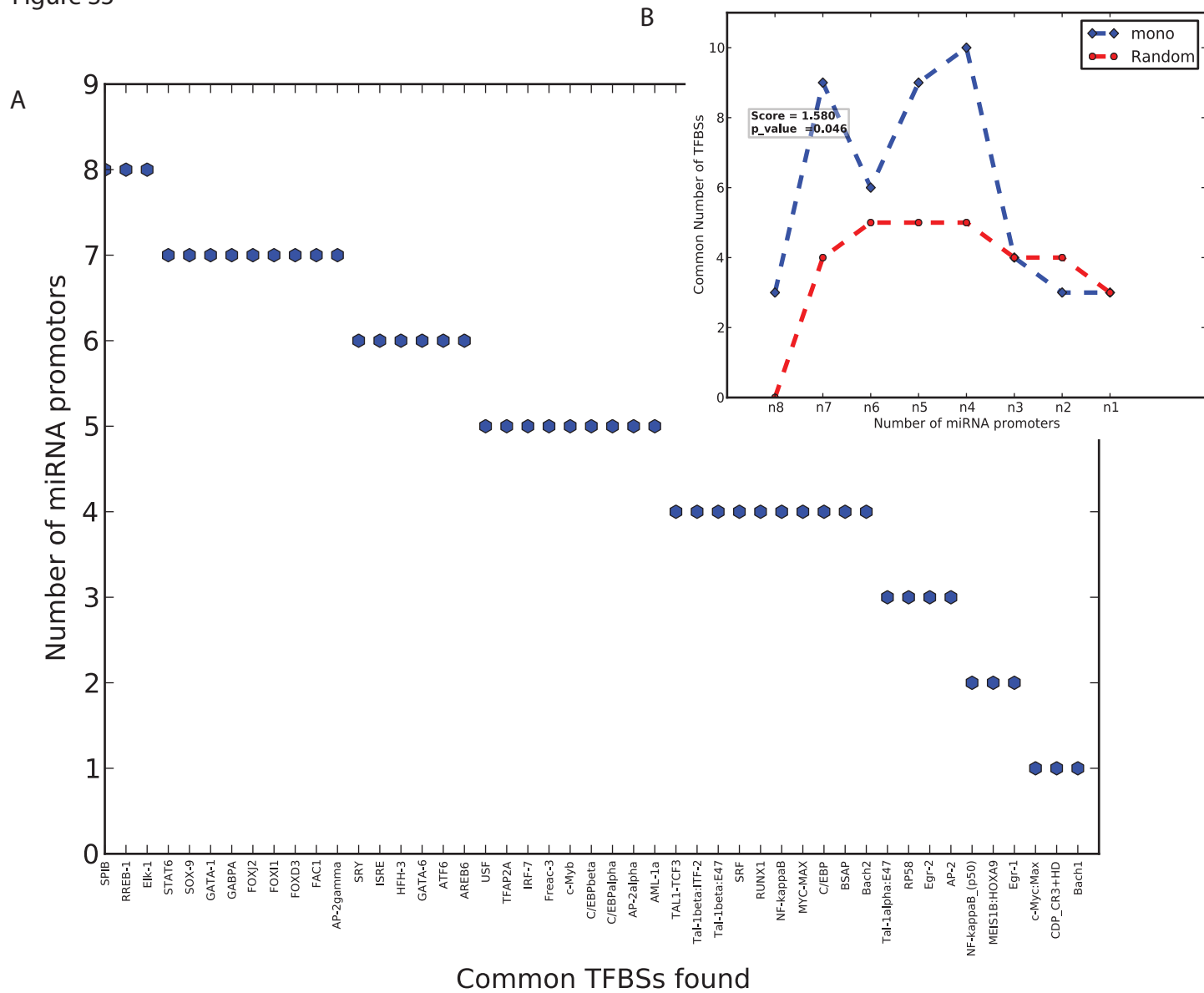

Supplement: Additional file 5 — Figure S3. (A) Commonality of TFBSs in promoter regions of monocyte-expressed miRNAs. Shown on the x-axis are the high-scoring TFBSs (i.e. of instance score ≥ 6) that occur at least once in the 2 kb promoters of the miRNA up-regulated in monocytes. The y-axis shows the number of promoters that have the TFBS, at least once at this threshold. (B) The distribution of the number of common TFBS hits per number of common miRNA promoters as in A, for test and random sets of miRNA promoters. The values for random are the median values from 1000 random set of miRNA promoters of same size and length as those in the monocyte set. The score is the ratio of the sum of "the product of numbers on the x-axis and corresponding y-axis values" for the monocyte set relative to that of the random. The p-value is the fraction of cases wherein this sum for random sets of miRNA promoters is greater than or equal to that of the DC set. [file 1471-2164-12-330-S5.PDF]

Figure S4

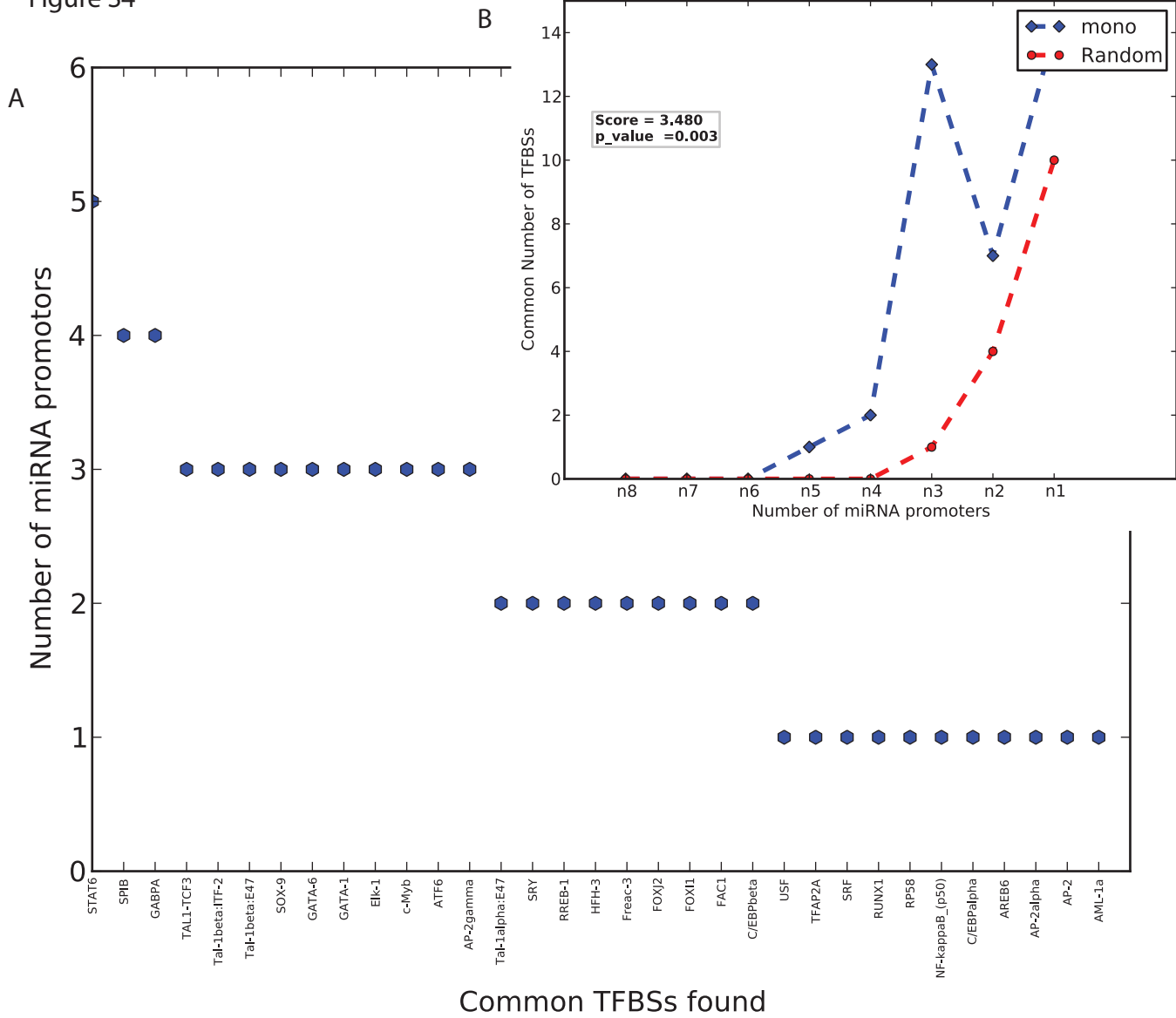

Supplement: Additional file 6 — Figure S4. (A) TFBSs shared among the promoter regions of miRNAs up-regulated in monocytes. Shown on the x-axis are high-scoring TFBSs filtered at a motif instance score threshold of atleast 6 and at a 10th percentile evolutionary conservation score that occur at least once in the promoters of miRNAs up-regulated in DCs. The y-axis shows the number of promoters that have the TFBS at least once at these thresholds. (B) The distribution of the number of common TFBS hits per number of common miRNA promoters as in A, for test and random sets of miRNA promoters. The values for random are the median values from 1000 random set of miRNA promoters of same size and length as those in the monocyte set. The score is the ratio of the sum of all occurrences of all TFBS for the monocyte set relative to that of the random sets. The p-value is estimated from the number of instances wherein this sum of "the product of number of TFBSs and the number of miRNA promoters" of 1000 random sets of miRNA promoters is greater than or equal to that of the monocyte set. [file 1471-2164-12-330-S6.PDF]

Figure S5

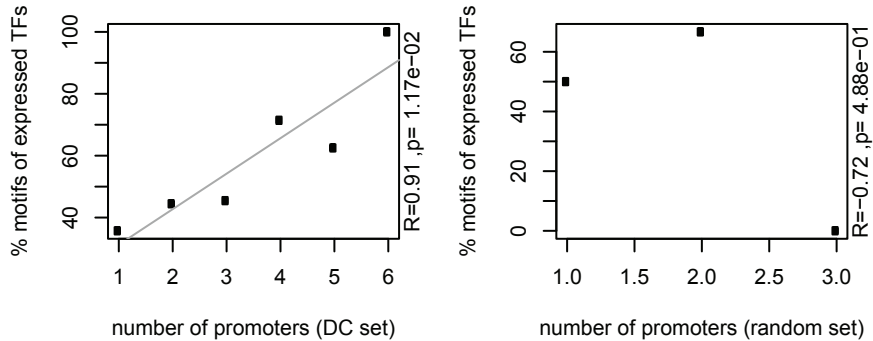

Supplement: Additional file 8 — Figure S5. Correlation of the number of expressed TFs with the number of promoters of miRNAs that are over-expressed in DCs. The TFs used are those of high-scoring TFBSs (i.e. of instance score > = 6) that are also highly conserved (10th percentile of PhastCons scores) and occur at least once in the 2 kb promoters of the miRNA up-regulated in DCs (left plot), and a random set of miRNAs (right plot). The original number of miRNA promoters that share at least one TFBS was 12 for both DCs and the random set of promoters (i.e the number of promoters of miRNAs that were over-expressed in DCs). Due to the high conservation threshold used, the maximum number of miRNA promoters that share at least one TFBS became smaller (6 for the test set and 3 for the random set). The correlations were calculated using the Pearson correlation as implemented in R. [file 1471-2164-12-330-S8.PDF]

Figure S6

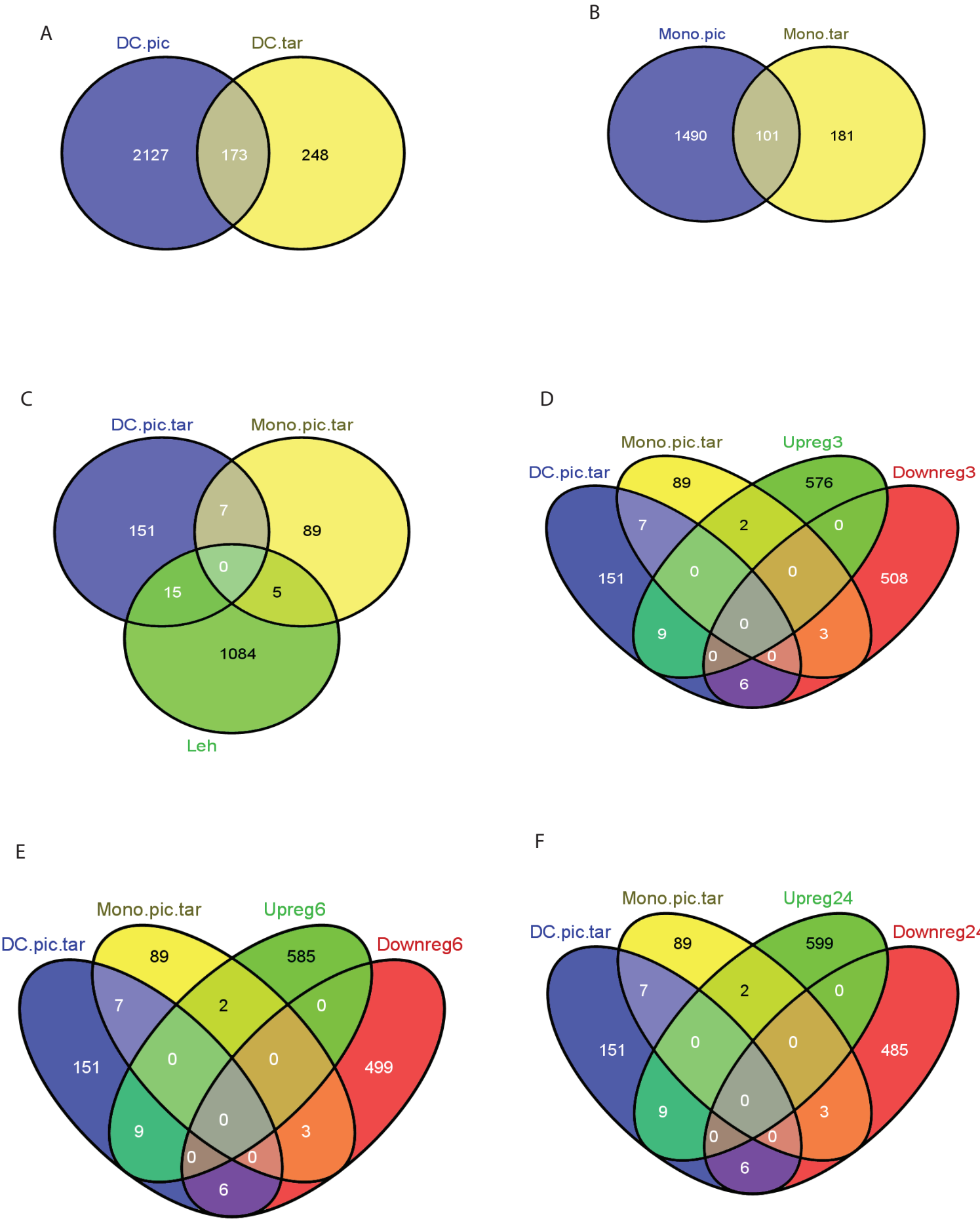

Supplement: Additional file 14 — Figure S6. Venn diagram showing the intersection of target genes. (A) Intersection of target genes of the miRNAs that are over-expressed in DCs, using PicTar (DC.pic), and TargetScan (DC.tar). (B) Similar to A, but for monocytes. (C) Intersections of the common targets found by PicTar and TargetScan for DC miRNAs (DC.pic.tar), the common targets for monocyte miRNAs (mono.pic.tar), and the dataset of Lehtonen et al. 2007 (Leh.) of genes that are regulated in DCs relative to monocytes during DC differentiation. (D-F) Intersection of common target genes from DC miRNAs, monocyte miRNAs, and genes that were up-regulated (upreg) or down-regulated (downreg), in DCs relative to monocytes in the data set of Lehtonen et al.2007. Figure D, E, F represent respectively the data sets at time points 3, 6 and 24 hr of DC differentiation from monocytes. (Differential gene relations (DC/monocyte) were selected at a fold change of at least 2. The PicTar score used > = 0.4, TargetScan context score < = -0.4. Comparisons were done at the level of RefSeq DNA ID and the elements in the intersections with the upreg and downreg datasets are provided in Table S11 with gene symbols attached. [file 1471-2164-12-330-S14.PDF]
